# Midostaurin reduces Regulatory T cells markers in Acute Myeloid Leukemia

**DOI:** 10.1038/s41598-018-35978-0

**Published:** 2018-12-03

**Authors:** Lucas Gutierrez, Miran Jang, Tian Zhang, Mojtaba Akhtari, Houda Alachkar

**Affiliations:** 1 School of Pharmacy, University of Southern California, Los Angeles, CA USA; 2 Norris Comprehensive Cancer Center, USC, Los Angeles, CA USA

## Abstract

Acute myeloid leukemia (AML) is a heterogeneous hematological malignancy in which the only curative approach is allogeneic stem cell transplant (Allo-HSCT). The recognition and elimination of leukemic clones by donor T-cells contribute significantly to Allo-HSCT success. *FLT3*-ITD, a common mutation in AML, is associated with poor prognosis. Recently, midostaurin became the first FDA approved FLT3-inhibitor for pre-transplant patients with *FLT3*-ITD in combination with standard therapy. In addition to their multikinase activity which may affect T-cell signaling, FLT3-inhibitors induce apoptosis of malignant cells which may also enhance antigen presentation to activate T-cells. Considering the increased clinical use of these inhibitors in patients with AML, and the limited clinical benefit derived from their use as single agents, understanding how FLT3-inhibitors affect T cell population and function is needed to improve their clinical benefit. We examined the effect of four different FLT3 inhibitors (midostaurin, sorafenib, tandutinib, and quizartenib) on T cell populations in peripheral blood mononuclear cells (PBMC) obtained from healthy donors and from patients with AML. Midostaurin exhibited a significant decrease in CD4 + CD25 + FOXP3+ T cell population and *FOXP3* mRNA expression in healthy and AML PBMCs. Similarly, samples collected from patients with AML treated with midostaurin showed a reduction in Tregs markers. Interferon-γ(IFN-γ), tumor necrosis factor-α(TNF-α), and IL-10 levels were also reduced following midostaurin treatment. Considering the FDA approval of midostaurin for use in patients with AML in the pre-transplant setting, our finding will have important clinical implication as it provides the rationale for functional investigation of the use of midostaurin in post-transplant patients.

## Introduction

Acute Myeloid Leukemia (AML) accounts for the highest mortality rate of all leukemias, and is the most common form of acute leukemia for adults in the United States^1^. AML is a heterogeneous hematologic malignancy characterized by clonal expansion of myeloid blasts in the blood and bone marrow^2^. The current therapies available for patients diagnosed with AML can vary according to the patient’s age and their disease risk status, however, the typical treatment options are induction therapy, consolidation therapy, and allogeneic hematopoietic stem cell transplant (Allo-HSCT)^3^. Of the treatments available, post-remission Allo-HSCT therapy is the only option which provides curative potential largely due to an immunological process called the graft-vs-leukemia (GvL) effect, in which donor cytotoxic T cells eradicate residual malignant cells^4^. However, Allo-HSCT may also result in a challenging condition called graft versus host disease (GvHD), wherein T cells target normal healthy cells, presenting a major toxicity to overcome^5^. Therefore, understanding the role of T cells in GvL is pivotal to optimizing patient’s treatment for AML.

Regulatory T cells (Tregs) are a subset of T cells that function in Allo-HSCT to maintain immune self-tolerance through suppression of aberrant or excessive immune responses that can be harmful to the patient^6^. One study demonstrated that the cotransfer of CD4 + CD25+ Tregs and CD4 + CD25− effector T cells into MHC-mismatched mice with leukemia prevented GvHD while preserving the beneficial GvL effect^7^. In addition, there is evidence that patients with AML receiving peripheral blood stem cell grafts with higher proportions of Tregs had a better 3-year survival rate compared with those receiving grafts with lower proportions of Treg populations^8^. Conversely, there is evidence for Treg inhibition of cytotoxic T lymphocytes and creation of an immunosuppressive or anti-apoptotic microenvironment that favors the survival of malignant hematopoietic cells^9^. Additionally, AML cells can influence the conversion of CD4+ CD25− cells into Tregs via tryptophan catabolism^10^. Tregs have been shown to suppress the T cell-mediated immune response against the leukemia cells by secretion of cytokines such as transforming growth factor β(TGFβ) or IL-10, and inhibiting dendritic cell maturation^11^.

One of the most common mutations in patients with AML is the FMS-like Tyrosine Kinase 3 receptor Internal Tandem Duplication (FLT3-ITD). The FLT3 receptor is expressed by immature hematopoietic progenitor cells and functions to induce proliferation and promote survival^12^. The ITD mutation alters the structure in the juxtamembrane domain of the FLT3 receptor, which leads to constitutive activation and continued proliferation of the AML cells. The *FLT3*-ITD mutation occurs in 30% of normal karyotype patients with AML and is associated with poor outcomes as well as increased incidence of relapse^13,14^. Although Allo-HSCT has significantly improved outcome of patients with AML, those positive for the *FLT3*-ITD mutation have a 50% chance of relapse within two years.

Due to the prevalence of the *FLT3*-ITD mutation in AML patients, targeting the FLT3 pathway through tyrosine kinase inhibitors has become a major focus in clinical efforts for developing novel therapeutic agents. Currently, a class of drugs called receptor tyrosine kinase inhibitors (TKIs) have been heavily investigated in patients with *FLT3*-ITD positive AML, and have demonstrated promising results in clinical trials. Very recently, the first FLT3 inhibitor midostaurin received FDA approval for treating patients with *FLT3*-ITD in combination with standard chemotherapy prior to Allo-HSCT^15,16^. However, one caveat of this novel therapeutic approach is that the inhibitors are not specific to the FLT3 receptor and may affect other signaling pathways, including those involved in T cell activation. In fact, previous *in vitro* studies have indicated that another TKI, sorafenib, may inhibit proper T cell function^17^. Therefore, if a T cell signaling pathway is affected in a way that can enhance the GvL effect, it is possible that TKIs can be used post-Allo-HSCT for therapeutic benefit. In this study, we examined the effect of four different TKIs sorafenib, midostaurin, tandutinib, and quizartenib on T cell populations in blood samples obtained from both healthy donors and patients with AML. Assessment of T cell populations, expression markers and cytokine levels showed that only midostaurin treatment significantly reduced the regulatory T cells population in the healthy and leukemic samples. These results indicate that further functional investigations are needed to establish whether midostaurin may have potential benefit or drawback if used in post-transplant setting.

## Materials and Methods

### Patient Samples

Blood samples were obtained from healthy donors or from patients with AML at diagnosis from Norris Comprehensive Cancer Center at USC. All samples were collected after obtaining written informed consent. The use of human materials was approved by the University of Southern California Health Sciences Campus Institutional Review Board in accordance with the Helsinki Declaration.

### Isolation of Peripheral Blood Mononuclear Cells (PBMCs)

PBMCs from Healthy donors were drawn and collected in sterile EDTA tubes (BD Vacutainer, Franklin Lakes, NJ). PBMCs were isolated by centrifugation over Ficoll-Paque PLUS density gradients (GE Healthcare, Uppsala, Sweden). Blood was diluted 1:2 in PBS, overlaid on Ficoll lymphocyte separation medium, and centrifuged at 400 × *g* for 30 minutes at room temperature. PBMCs were collected and washed twice with phosphate buffered saline (Sigma, St. Louis, MO). The final pellet was resuspended in 20% FBS RPMI, and used in the proceeding experiments.

### Cell Culture

PBMCs were isolated as previously stated. Immediately after isolation, PBMCs were cultured in Roswell Park Memorial Institute 1640 1x (RPMI) medium supplemented with 20% fetal bovine serum (FBS) (Gibco, Gaithersburg, MD). In addition, PBMCs were treated with IL-2 (5 ng/mL) and IL-7 (10 ng/mL) (Life Technologies, Carlsbad, CA), as well as 1 µM of either Sorafenib, Midostaurin, Tandutinib, or Quizartenib. PBMCs were seeded at 2 million cells per well in a 6 well plate (Genesee Scientific, San Diego, CA), and allowed to incubate at 5% CO_2_, 37 °C for 72 hours before analysis via flow cytometry or quantitative polymerase chain reaction.

### RNA Extraction

Cells were centrifuged at 1300 rpm for 5 minutes, supernatant was removed, and pellet was resuspended in 500 μl of TRIzol reagent and allowed to incubate at room temperature for 2.5 minutes. Subsequently, 200 μl of Chloroform was added to each sample and shaken vigorously for 15 seconds and allowed to incubate on ice for 15 minutes. Samples were then centrifuged at 15000 × *g* for 15 minutes at 4 °C. The upper aqueous phase was then transferred to an RNAse-free 1.5 mL Eppendorf tubes. 200 μl of isopropanol was added to the samples, and incubated on ice for 10 minutes. Then, samples were centrifuged at 15000 × *g* for 15 minutes at 4 °C. Once complete, the pellet was washed with 75% ethanol, and centrifuged at 15000 × *g* for 15 minutes at 4 °C. The supernatant was discarded. The pellet was air-dried for 5 minutes, and resuspended in 20 μl of DEPC-treated ddH_2_0.

### Quantitative Polymerase Chain Reaction (qPCR)

cDNA synthesis was performed with random hexamer primers using the Superscript first strand synthesis system for qPCR (Invitrogen, Carlsbad, CA). qPCR was carried out using the 7500 Real Time PCR system and Taqman assays from Applied Biosystems for analysis of *B2M*, *FOXP3*, and *Granzyme B*. Expression of genes of interest were normalized to *B2M*, the house-keeping gene. Subsequently, expression was normalized to that of the negative control sample.

### Flow Cytometry Analysis

The T cell population from PBMCs were analyzed via FACS after 72 hours of treatment with tyrosine kinase inhibitors and IL-2/IL-7. T cell populations were identified through PerCP-labelled anti-CD3 (eBioscience, San Diego, CA); specific T cell subpopulations were identified with PE-labelled anti-CD4 (eBioscience, San Diego, CA), PE-Cyanine7-labelled anti-CD8 (eBioscience, San Diego, CA), APC-labelled anti-CD25 (eBioscience, San Diego, CA), and FITC-labelled anti-Foxp3 (eBioscience, San Diego, CA). Data was acquired using the BD LSRII Flow Cytometer (BD Biosciences, San Jose, CA), and analyzed using FACS Diva software (BD Biosciences, San Jose, CA). Cells were gated for CD3+, then CD4+, then the percentage of CD4+ CD25+, and finally the percentage of CD4+CD25+Foxp3+ populations were quantified.

### Cytokine Measurement via Meso Scale Discovery Assay

Supernatants from PBMCs treated with tyrosine kinase inhibitors were stored at −80 °C for analysis. Cytokine ELISAs were performed using electrochemiluminescent multiplex assays to determine the p levels of four cytokines (IFN-γ, TNF-α, IL-10, TGFβ). Calibration curves were prepared in the supplied assay diluents, with a range of 17500 to 0.93 pg/ml. Cytokine concentrations were determined with MSD Workbench 3.0 software (Meso Scale Discovery, Gaithersburg, MD, USA), using curve fit models (log- log or four-parameter log-logistic).

### Cell Viability

Cell viability was determined by Alamar Blue–based metabolic assay according to the manufacturer’s instructions (Invitrogen, Carlsbad, CA). At 0 and 72 hours, 10 μl of Alamar Blue reagent was added to each well containing 90 μl of resuspended cells; and absorbance (ΔOD_570 nm–600 nm_) was measured on an automated 96-well spectrophotometer after color development. In addition, cell viability was also determined by Trypan Blue stain.

### Statistics

The data are presented as mean ± standard error (SE). The Student *t* test was used to determine if the difference in mean between samples was statistically significant: *p* < 0.05 was considered significant.

### Ethics approval and consent to participate

This study was conducted according to the approved IRB protocol.

## Results

### Midostaurin Reduces CD4 + CD25 + FOXP3 + T cell Population in healthy PBMC

**Figure 1:**
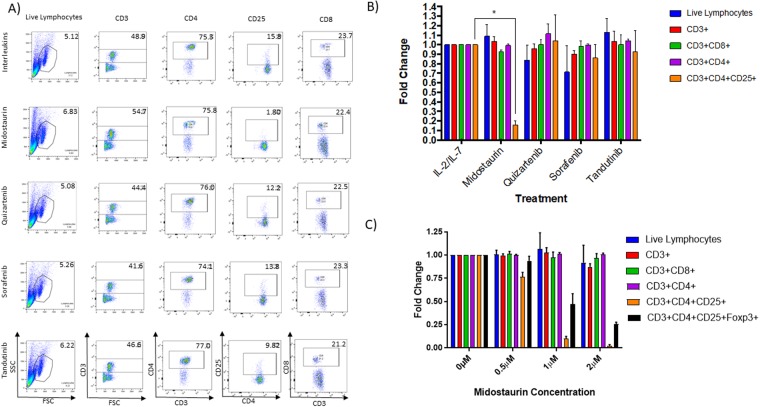
Midostaurin reduces CD4 + CD25+ population in healthy PBMCs. (**A**) Representative contour plots of T cell populations from Healthy donors treated with inhibitors. (**B**) *In vitro* PBMCs were treated with 4 kinase inhibitors and normalized to IL-2/IL-7 control population percentages (N = 3, p < 0.05). (**C**) Combined quantification of multiple samples of PBMCs treated with 0.5, 1 and 2uM of midostaurin and normalized to IL-2/IL-7 control population percentages (N = 3, p < 0.001).

**Figure 2:**
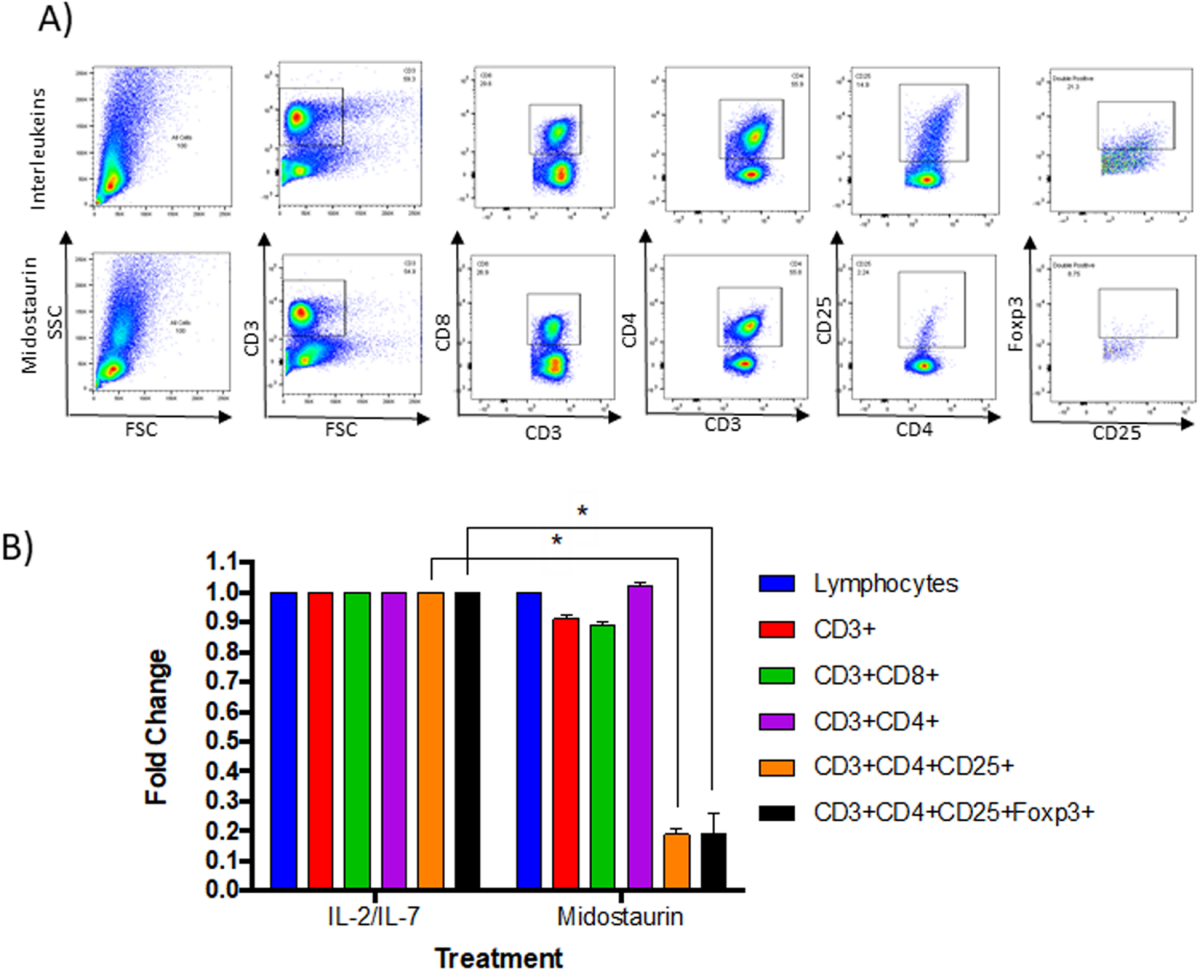
Midostaurin reduces CD4 + CD25 + FOXP3+ population in healthy PBMCs. *In vitro* PBMCs were treated with 1μM midostaurin, T cell populations were analyzed by flow cytometry (**A**) a representative figure. (**B**) Normalized to IL-2/IL-7 control population percentages and averaged (N = 3, p < 0.001).

We isolated PBMCs obtained from healthy volunteers and from patients with AML. We treated cells for 72 hours with either sorafenib, midostaurin, tandutinib, or quizartenib, each at concentration of 1μM, combined with IL-2 (5 ng/ml) and IL-7 (10 ng/ml). We used 1μM dose, which is significantly higher than the IC50 calculated for each inhibitor in *FLT3*-ITD positive AML cells (MV4-11), but not effective in T cells (T ALL cell lines: MOLT4 and RPMI8402) (Fig. S1), and is still achievable in blood of patients with AML. T cell populations (CD3+, CD4+, CD8+ and CD4 + CD25+ cells) were evaluated by flow cytometry analysis (Fig. 1A). We found that PBMCs from Healthy donors treated with midostaurin had a statistically significant decrease in mean CD4+ CD25+ T cell population when compared with other treatment groups (N = 3, 80% decrease, P < 0.001, Fig. 1B). Sorafenib showed a modest decrease in mean CD4 + CD25+ T cells. On the other hand, treatment with tandutinib and quizartenib did not affect CD4 + CD25+ population. No effect was observed on total lymphocytes, CD3+, CD8+, and CD4+ T cells, when cells were treated with FLT3 inhibitors (Fig. 1B). We also treated healthy PBMC from three different donors with increasing concentrations of midostaurin (0.5 μM, 1 μM, and 2 μM), and observed a dose response effect of midostaurin on CD4 + CD25 + FOXP3+ cells (Fig. 1C). Additionally, intracellular staining of FOXP3 demonstrated a significant decrease of T cells in the midostaurin-treated group compared with the control group (n = 3, 70–90% decrease, P < 0.001, Fig. 2).

### Midostaurin Reduces *FOXP3* mRNA expression and Modulates T cell Cytokine Activity in healthy PBMC

**Figure 3:**
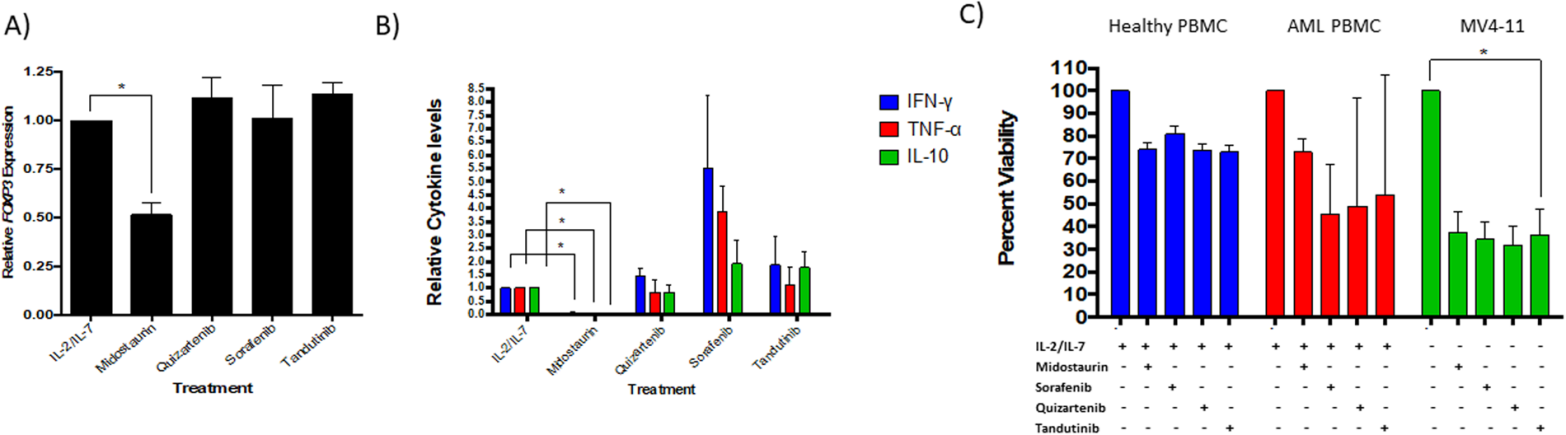
Midostaurin Reduces *FOXP3* Expression and T cell cytokines. (**A**) Effect of various inhibitors on *FOXP3* mRNA expression in healthy PBMCs (N = 5, p < 0.05). (**B**) Cytokine levels in picograms/ml were measured in cell treated supernatants and normalized to IL-2/IL-7 treated group (n = 3 p < 0.05). (**C**) PBMCs were treated with four different kinase inhibitors for 72 hours and showed a 20% reduction in viability for both healthy (N = 3) and AML (N = 5) groups. MV4-11 cells were used as a positive control for kinase inhibitors and showed over 50% reduction in viability (N = 8).

Treatment with midostaurin resulted in a statistically significant decrease in relative *FOXP3* mRNA expression compared with other treatment groups of the healthy PBMCs (N = 5, 2-fold decrease, P = 0.02, Fig. 3A). On the other hand, sorafenib, tandutinib, and quizartenib did not affect *FOXP3* mRNA levels. Then, we measured the levels of interferon-γ (IFN-γ), tumor necrosis factor-α (TNF-α), and IL-10 in the supernatants of treated cells and compared it with untreated cells. We found that midostaurin treatment resulted in significant decrease in IFN-γ, TNF-α, and IL-10 (N = 3. 95–98% decrease, P < 0.001 levels, Fig. 3B). Sorafenib showed an increase in IFN-γ (N = 3, 5-fold increase, P = 0.04), TNF-α (N = 3, 4-fold increase, P = 0.02), and IL-10 (N = 3. 2-fold increase, P = 0.17) levels (Fig. 3B). None of the kinase inhibitors exhibited significant effect on the TGFb levels compared with that of the control groups (Fig. S3A). The effects of different treatments on cell viability are shown in Fig. 3C. T cells express very low level of *FLT3* compared with myeloid leukemic cells (Fig. S2, n = 5 for T cells, n = 3 MV4-11 controls, p < 0.001). Thus, the effect of midostaurin on T cell markers is likely a FLT3-independent effect.

### Midostaurin-treated PBMCs from Patients with AML Display a Decrease in Regulatory T cells

**Figure 4:**
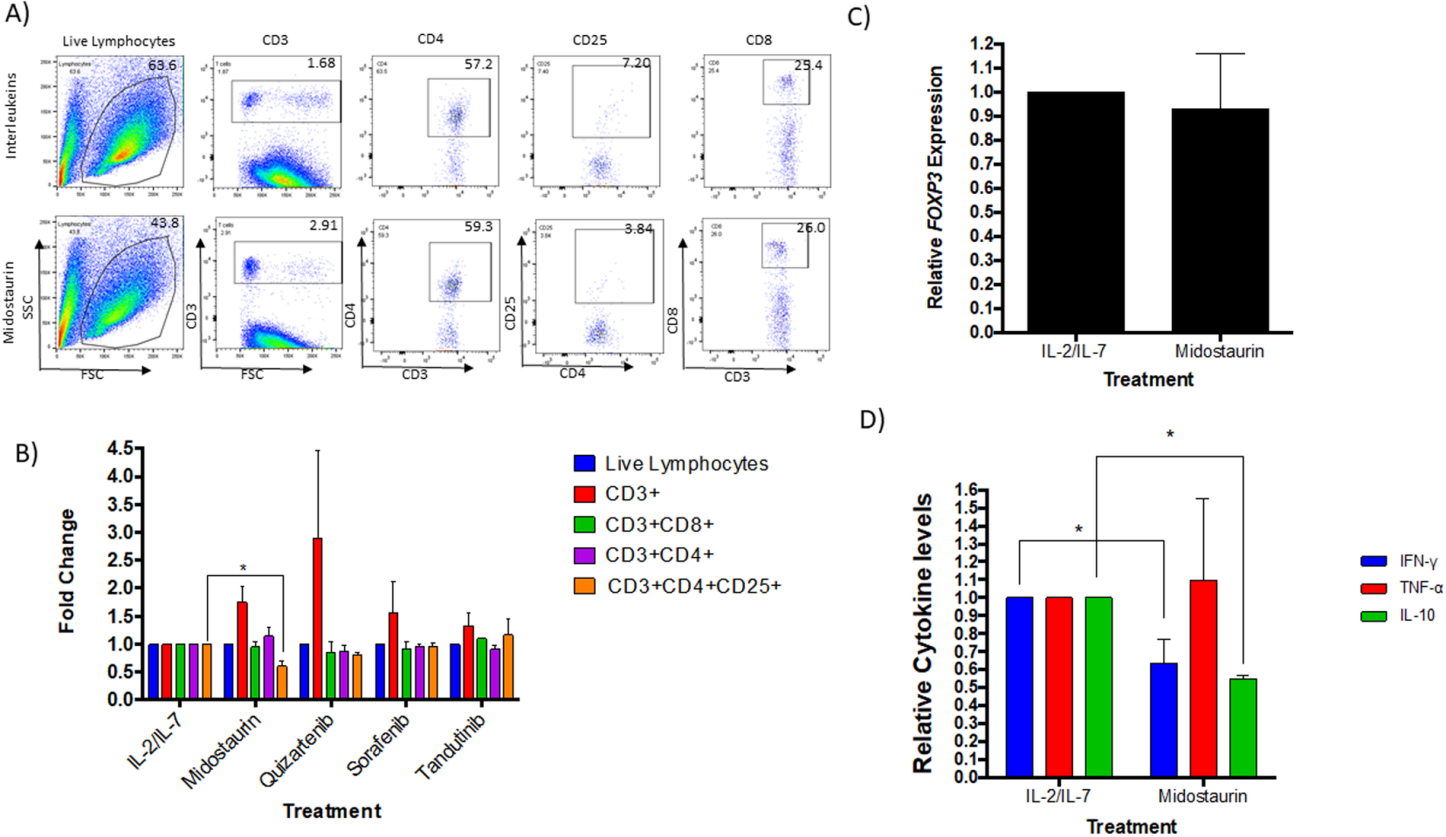
Midostaurin reduces Tregs in AML cells. (**A**) Representative contour plots of T cell populations from patients with AML treated with midostaurin. (**B**) *In vitro* PBMCs were treated with one of the four kinase inhibitors and normalized to IL-2/IL-7 control population percentages (n = 4, p < 0.05). (**C**) *FOXP3* mRNA expression in AML PBMCs (n = 4) treated *ex vivo* with 1uM of midostaurin. (**D**) Cytokine levels in picograms/ml were measured in AML cells treated supernatants and normalized to IL-2/IL-7 treated group (N = 5).

To validate these findings in AML; PBMCs isolated from blood of patients with AML were treated with midostaurin for 72 hours in the presence of IL-2 and IL-7, and compared with untreated cells. Our initial results, obtained via flow cytometry (Fig. 4A)-found a significant decrease in the CD4 + CD25+ T cell population (N = 6, 30% decrease, P = 0.001, Fig. 4B) in midostaurin treated samples compared with their respective controls. PBMCs extracted from patients with AML and treated with midostaurin also displayed a slight decrease in *FOXP3* mRNA expression compared to the untreated PBMCs; however, the difference was not statistically significant (N = 6, 4–55% decrease, P = 0.30, Fig. 4C). In addition, cytokine analysis of supernatants of AML-PBMCs treated with midostaurin showed a change in IFN-γ (N = 5, 25% decrease, P = 0.002), TNF-α (N = 3, 10% increase, P = 0.42), and IL-10 (N = 2, 50% decrease, P = 0.001) when compared to the control group (Fig. 4D). IL-10 levels were below the detection level of our assay in three patient’s samples. Also, similar to the healthy PBMCs, TGFβ levels were not significantly changed following midostaurin treatment of AML samples (Fig. S3B). Viability assay confirmed that treatment with FLT3 inhibitors decreased cell viability in AML PBMCs by about 17–33%, however, treatment with FLT3 inhibitors decreased cell viability of MV4-11 cells (an AML cell line that carries the *FLT3*-ITD mutation) by more than 50% (Fig. 3C). Although the percentage of T cells was very low in the blood obtained from the diagnostic samples obtained from patients with AML, the observed trend was similar to that found in healthy PBMC.

### Midostaurin reduces Tregs markers in patients with AML

**Figure 5:**
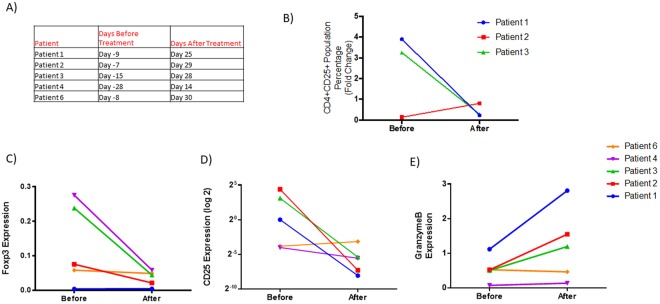
Midostaurin alters CD4 + CD25 + T cell population and T cell gene expression markers in patients with AML. (**A**) Table indicating time points before and after midostaurin treatment for patient samples. (**B**) Levels of CD4 + CD25+ population before and after midostaurin treatment. (**C**) *FOXP3* and (**D**) *CD25* mRNA normalized to *CD3* and *CD4* expression before and after midostaurin treatment. (**E**) Expression change of *GZMB* mRNA normalized to *CD3* and *CD8* mRNA expression before and after midostaurin treatment.

Because midostaurin is currently FDA approved for treatment of AML in combination with chemotherapy, we validated our findings in patients who received midostaurin. We obtained samples from three patients with AML at sequential times before and after midostaurin treatment. CD4 + CD25+ cells population decreased approximately four weeks after the initiation of midostaurin treatment compared with time points before the treatment in two patients (Fig. 5B). In one patient, CD4 + CD25+ cells were already low at base line and no change was observed following midostaurin treatment. We also assessed *FOXP3*, *CD25 and GranzymeB* mRNA levels in five patients. We found a consistent decrease in *FOXP3* and *CD25* levels and increase in GranzymeB levels in three out of five patients, the two patients where no significant change observed also had very low base level of *FOXP3* and *CD25* mRNA before treatment (Fig. 5C–E).

## Discussion

FLT3 and FLT3 ligand (FLT3L) signaling has been shown to indirectly expand Treg through increasing dendritic cell number^18^. Therefore, it is plausible that mechanisms responsible for activating FLT3 signaling pathways may also cause an expansion in regulatory T cells and thus induce a repressive immune response and immune evasion in leukemic cells. As a result, we hypothesized that inhibiting FLT3 signaling pathways with FLT3 inhibitors would affect T cell populations and particularly regulatory T cells. Unlike the second generation FLT3 inhibitors, sorafenib and quizartinib, the first generation FLT3 inhibitors midostaurin and tandutinib are less specific and inhibit a wide range of tyrosine kinases. Unexpectedly, only midostaurin but not every FLT3 inhibitor resulted in the significant decrease in Tregs both in healthy PBMCs and AML PBMCs. This suggests that mechanisms other than those mediated by FLT3 signaling pathways are responsible for this reduction in the Treg. Previous studies have demonstrated that IL-2 and IL-7 are important for maintaining homeostasis of Treg cells^19,20^. IL-7 induces the expression of CD25 on the surface of T cells and expand Treg^21,22^. Also, IL-2 has been shown to increase the CD4 + CD25+ T cell population in cancer patients by 4-fold *in vivo*, as well as increase their suppressive capabilities *in vitro*^23^. In addition, there is evidence that induced Tregs (iTregs) require a constant supply of IL-2 for proper development and function^20^. Whether midostaurin interferes with IL-2 and IL-7 signaling pathways and downstream targets is unclear. Midostaurin inhibitory effects on JAK/STAT and PI3K/AKT signaling pathways which are also downstream of IL-2 and IL-7 may potentially be a plausible mechanism. Previous studies have demonstrated that PI3K/AKT inhibitors can decrease Treg populations without affecting other T cell populations^24^. STAT5 is crucial for IL-2 response, Treg cell development, and for the expression of *FOXP3*^25–27^. In fact, STAT5 is a transcription factor that also functions downstream of FLT3-ITD, making it a potential target for midostaurin^28^. Recent studies have reported that tyrosine kinase inhibitors increase the cell surface localization of FLT3-ITD^29^. Although the study did not address the effect of FLT3 inhibition on regulatory T cells, this suggests a dual effect of midostaurin in enhancing FLT3-directed immunotherapy of AML. Although the mechanism by which midostaurin reduces Tregs is not clear, our data indicates possible off-target effects of this multi-kinase inhibitor on T cell signaling pathways that is differentially specific to Treg cells. A limitation of the study is the lack of comprehensive profiling of T cell markers, in order to exclude potential effects of midostaurin on other T cell populations. We are currently exploring the effect of midostaurin on Tregs function and repertoire, as this may provide valuable information on improving GvL effect in patients with AML.

We conclude that midostaurin demonstrated a decrease in Treg populations both *in vitro* and in some patients with AML. In light of the recent FDA approval of midostaurin combined with chemotherapy in patients with AML^16^, these results highlight a novel therapeutic advantage of this drug that may be beneficial particularly in the post-transplant setting as well as in combination with immunotherapy.

## Electronic supplementary material

Supplemental data

## Data Availability

The datasets used and/or analyzed during the current study are available from the corresponding author on reasonable request.
