# Supplementary material for: Midostaurin reduces Regulatory T cells markers in Acute Myeloid Leukemia
Source: Sci Rep. 2018 Dec 3;8:17544. doi: 10.1038/s41598-018-35978-0 (PMC6277419; doi:10.1038/s41598-018-35978-0)
Supplement: Supplementary file 1 — Supplemental data [file 41598_2018_35978_MOESM1_ESM.pdf]

Midostaurin reduces Regulatory T cells markers in Acute Myeloid Leukemia

Authors: Lucas Gutierrez[1], M.S., Miran Jang[1], PhD, Tian Zhang[1], M.S., Mojtaba Akhtari[2], MD, Houda Alachkar[1,2], PhD, PharmD

Supplemental figures and tables.

Supplemental Tables:

Table S1. Cytokine levels in supernatants of PBMCs from healthy patients after stimulation with midostaurin for 72 hours

	IL-10		IFN		TNF		TGFB	
	IL-2/IL-7 Control	Midostaurin Treatment	IL-2/IL-7 Control	Midostaurin Treatment	IL-2/IL-7 Control	Midostaurin Treatment	IL-2/IL-7 Control	Midostaurin Treatment
Donor 1	4.20	0.14	301.62	37.59	54.50	1.34	1711.09	1422.20
Donor 2	3.13	0.11	8043.09	32.35	250.95	1.57	2103.83	2891.72
Donor 3	8.55	0.06	1819.51	26.46	68.47	1.14	2454.79	1612.67

Table S2. Cytokine levels in supernatants of PBMCs from patients with AML after stimulation with midostaurin for 72 hours

	IL-10		IFN		TNF		TGFB	
	IL-2/IL-7 Control	Midostaurin Treatment	IL-2/IL-7 Control	Midostaurin Treatment	IL-2/IL-7 Control	Midostaurin Treatment	IL-2/IL-7 Control	Midostaurin Treatment
AML sample1	1.29E-04	0.01	23.72	18.79	0.19	0.23	917.62	834.24
AML sample 2	0.01	0.01	23.22	19.03	0.81	0.34	920.06	827.42
AML sample 3	5.44	2.92	72.46	44.30	3.90	3.07	1690.31	1843.87
AML sample 4	0.02	0.03	24.50	20.59	0.99	1.98	2039.02	1861.33
AML sample 5	41.96	23.77	2450.20	283.33	7506.45	3819.59	1748.25	1652.53

Table S3: Clinical information related to patients treated with midostaurin included in the study.

Patient #	Age	Diagnosis	% PB blasts	% BM blasts	FLT3 status	Treatement with midostaurin	Transplant Status	Months Post-Transplant	Treatment Outcome	Survival-Months	Survival Status
Patient 1	75	newly diagnosed AML	85%	>90%	FLT3-ITD	Cytarabine-midostaurin at induction	no	n/a	complete remission	12 months	alive
Patient 2	48	post transplant relapsed AML	58%	>80%	FLT3-ITD	Cladribine-Cytarabine-midostaurin at induction	yes	27	complete remission	1.5 months	deceased (disease unrelated cause)
Patient 3	37	newly diagnosed AML	57%	60%	FLT3-ITD	midostaurin starting 3 months post transplant	yes	4	complete remission	11.5 months	alive
Patient 4	71	newly diagnosed AML	65%	94%	FLT3-TKD	consolidation AraC with midostaurin	no	n/a	complete remission	6.5 months	alive
Patient 6	41	newly diagnosed AML	rare(>1%)	26%	FLT3-ITD	consolidation cytarabine/daunorubicin with midostaurin	yes	3	complete remission	5.5 months	alive

Supplemental Figures:

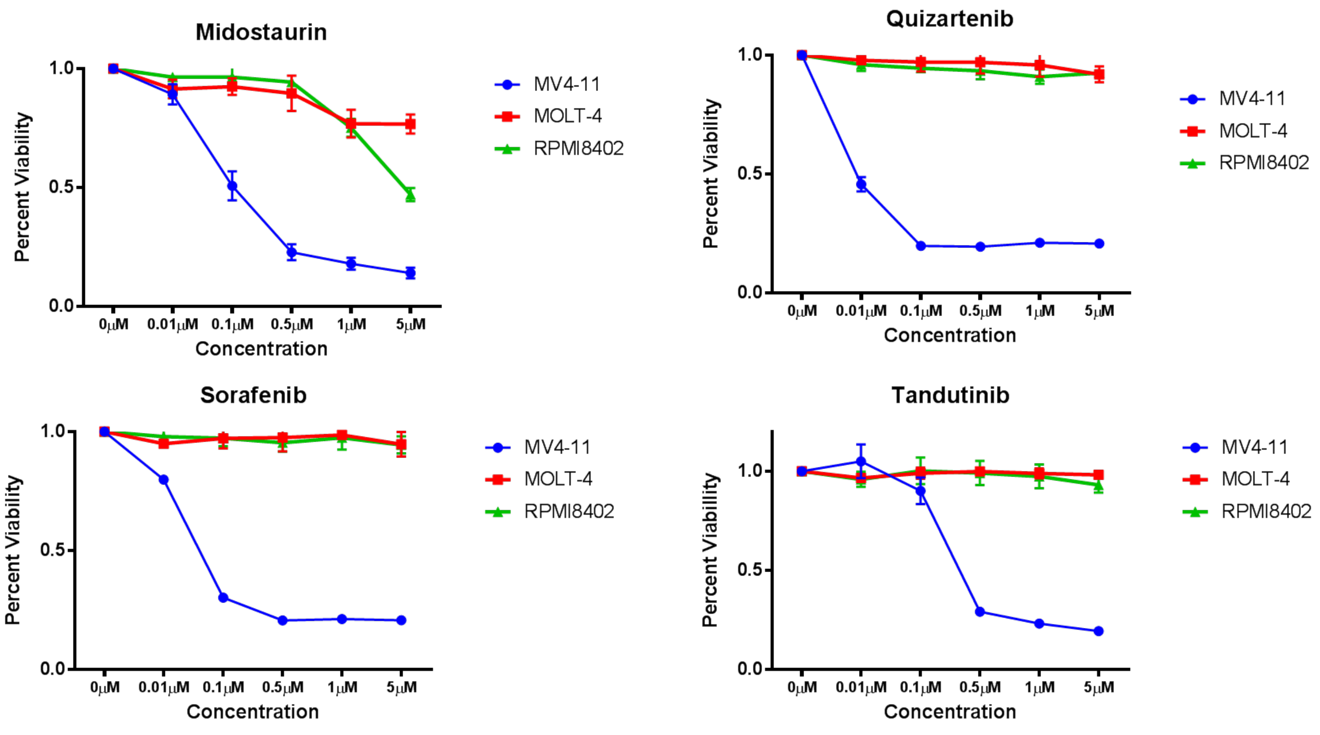

<u>Figure S1:</u> IC50 of four FLT3 Inhibitors in MV4-11, RPMI8402, and MOLT-4 for A) Midostaurin, B) Quizartenib, C) Sorafenib, D) and Tandutinib (N=3).

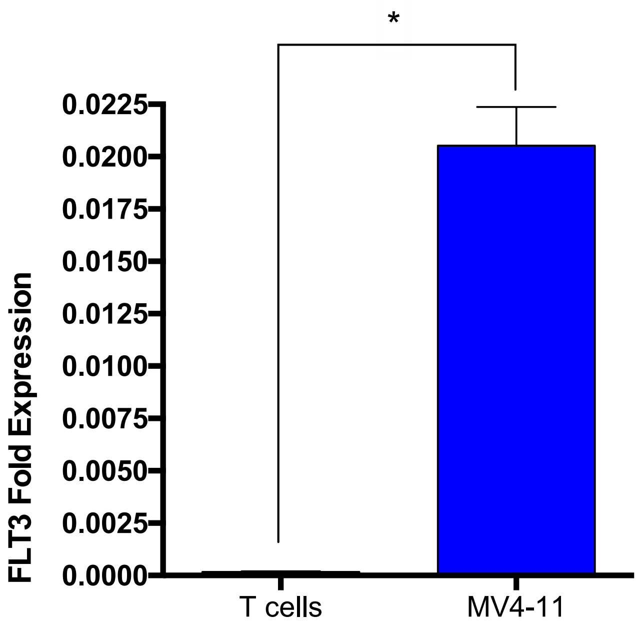

Figure S2. FLT3 Expression in T cells. Levels of FLT3 were measured in T cells (N=5) and compared with MV4-11 cells (n=3)(P<0.001).

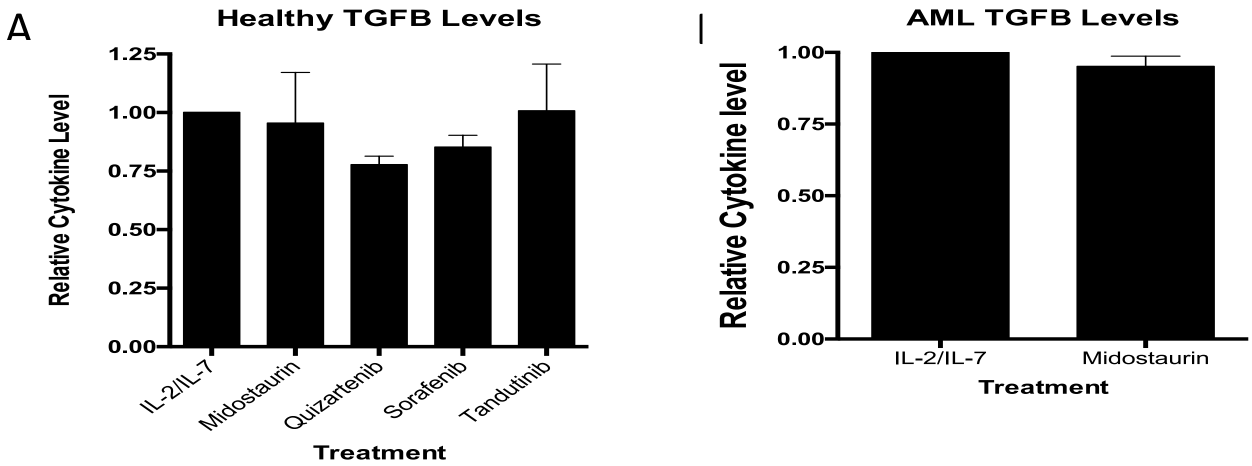

Figure S3. Midostaurin does not affect TFGβ levels. A) TGFβ levels in picograms/ml were measured in cell treated supernatants and normalized to IL-2/IL-7 treated group for healthy patients (n=3, p=0.42) B) TFGβ levels in picograms/ml were measured in cell treated supernatants and normalized to IL-2/IL-7 treated group for patients with AML (n=5, p=0.10).
